# Slice Cultures as a Model to Study Neurovascular Coupling and Blood Brain Barrier In Vitro

**DOI:** 10.1155/2011/646958

**Published:** 2011-02-16

**Authors:** Richard Kovács, Ismini Papageorgiou, Uwe Heinemann

**Affiliations:** Institute for Neurophysiology, Charité-Universitätsmedizin Berlin, Oudenarder Strasse 16, 13347 Berlin, Germany

## Abstract

Proper neuronal functioning depends on a strictly regulated interstitial environment and tight coupling of neuronal and metabolic activity involving adequate vascular responses. These functions take place at the blood brain barrier (BBB) composed of endothelial cells, basal lamina covered with pericytes, and the endfeet of perivascular astrocytes. In conventional *in vitro* models of the BBB, some of these components are missing. Here we describe a new model system for studying BBB and neurovascular coupling by using confocal microscopy and fluorescence staining protocols in organotypic hippocampal slice cultures. An elaborated network of vessels is retained in culture in spite of the absence of blood flow. Application of calcein-AM either from the interstitial or from the luminal side resulted in different staining patterns indicating the maintenance of a barrier. By contrast, the ethidium derivative MitoSox penetrated perivascular basal lamina and revealed free radical formation in contractile cells embracing the vessels, likely pericytes.

## 1. Introduction

Proper function of the central nervous system requires a meticulously controlled interstitial environment. Since its composition largely differs from that of blood plasma, its maintenance relies on selective filtering and active transport processes at the blood brain barrier (BBB). In order to keep pace with the energetic demand of neuronal activity, cerebral blood flow is tightly regulated by multiple and only partially understood mechanisms termed as neurovascular coupling. The structural substrate for BBB and neurovascular coupling is the neurovascular unit composed of tight junction coupled endothelial cells, capillary basal lamina covered with smooth muscle cells (SMCs)/pericytes, and the endfeet of perivascular astrocytes [1]. 

Studies on BBB and neurovascular coupling are frequently done *in vivo* although the exact control of systemic effects is difficult. Accordingly, the conclusions which can be drawn need careful interpretation. Studies on acute brain slices gave us new insights on the regulation of capillary microcirculation [2–5] as well as on consequences of BBB disruption [6, 7]. However, brain slices represent acutely injured tissue with severed BBB and ongoing cell damage that might negatively interfere with the mechanisms of neurovascular coupling [8, 9].

Widely used *in vitro* models of BBB are based on different cocultures of endothelial cells and astrocytes [10]. However, such models dismiss the intimate influence of the surrounding nervous tissue, pericytes, and perivascular microglia on the development and function of BBB.

Organotypic brain slice cultures [11, 12] gained popularity after the invention of Stoppini's method of culturing on a membrane surface [13]. Although slice cultures retain the cellular diversity of the CNS, most of the studies focused exclusively on the neuronal compartment. One of the few exceptions was a promising approach for modeling BBB by cultivating brain slices on top of confluent endothelial cell cultures [14, 15]. We wondered whether functional and structural properties of the neurovascular unit and BBB are maintained within slice cultures and thus offer the possibility to study neurovascular coupling and transport processes at the BBB *in situ*.

Moser and colleges were the first to describe the survival of endothelial cells and vessel-like structures in organotypic slice cultures from rat cortex [16]. More recently, intactness of basal laminae, expression of structural components like tight junction and transport proteins as well as ensheathment of the vessels by GFAP positive astrocytes were demonstrated by immunofluorescence in slice cultures from mice [17, 18]. Thus, structural criteria of BBB seem to be fulfilled in this preparation. 

Here we sought to characterize the functional intactness of the neurovascular unit and BBB in hippocampal slice cultures. We developed fluorescent staining protocols allowing for selective labeling of perivascular astrocytes, SMCs, pericytes, and endothelial cells in parallel with measurements of intracellular calcium concentration ([Ca^2+^]*_i_*) in astrocytes, as well as of contraction and reactive oxygen species (ROS) formation in pericytes or in SMCs. We used a combination of bulk and bolus staining methods taking advantage of the selective permeability of the BBB for different dyes.

## 2. Materials and Methods

Slice cultures were prepared and maintained as described previously [19]. Briefly, 7- to 8-day-old Wistar rat pups were decapitated, the brains were removed and submerged in ice-cold minimal essential medium (MEM) gassed with carbogen (95% O_2_, 5% CO_2_). Hippocampal slices (400 *μ*m, McIllwain Tissue Chopper, Mickle Laboratories, Guildford, UK) were cut and placed on a culture plate insert (MilliCell-CM, Millipore, Eschborn, Germany). Slice cultures were used for experiments between 3 to 21 days *in vitro*. Culture medium (containing: 50% MEM, 25% Hank's Balanced Salt Solution, 25% Horse Serum, pH 7.4; all from Gibco, Eggenstein, Germany) was replaced three times a week.

Slice cultures were transferred to the recording chamber mounted on an epifluorescent microscope (Olympus BX51WI, Olympus-Europe GmbH, Hamburg, Germany) and were superfused with ACSF (5 mL/min, 30°C), containing (in mM): NaCl 129, KCl 3, NaH_2_PO_4_ 1.25, MgSO_4_ 1.8, CaCl_2_ 1.6, NaHCO_3_ 26, and glucose 10 (pH 7.4). For induction of epileptiform activity, Mg^2+^ was omitted from the perfusion and [K^+^]*_o_* was slightly elevated to 5 mM.

Local field potential recordings were performed in area CA3 of slice cultures by using a MultiClamp 700B amplifier (Axon CNS, Molecular Devices, Sunnyvale, California, USA). Fluorescence recordings were performed with a spinning disk confocal microscope (Andor Revolution, BFIOptilas GmbH, Gröbenzell, Germany) equipped with an EMCCD camera (Andor iXonEM+) and a PIFOC fast-piezo z-scanner (Physik Instrumente, Berlin, Germany). Fluorescence was obtained by a 60x water immersion objective (N.A.: 0.9), laser intensity below the objective was below 10 *μ*W for the 491 nm and <50 *μ*W for the 561 nm laser line. In order to minimize photobleaching and interaction of illumination with ROS formation, exposure time was kept short (80–120 ms) and acquisition rate for time series reduced to one z-scan per 3 s for rhod-2 and one z-scan per 20 s for calcein/MitoSox. To avoid movement artifacts due to tissue swelling and apparent movement of the vessels, for each time point 8–12, z-planes were obtained (0.6–1.2 *μ*m steps), containing the whole vessel. At the beginning and at the end of an experiment, a high resolution z-scan (0.3 *μ*m steps) was acquired for volume rendering and 3D reconstruction of the vessels and surrounding astrocytes.

Slice cultures were bulk stained either with MitoSox, (5 *μ*M, 10–60 min) or rhod-2 AM (5 *μ*M, 10–60 min) and calcein-AM (4 *μ*M, 10–60 min) in the incubator at 36°C. All dyes were prepared in DMSO (0.1% in final solution) immediately before each experiment. A bolus of calcein-AM (400 *μ*M, freshly diluted in ACSF from the 4 mM DMSO stock) was applied either in the vicinity of the vessel or into the vessel lumen via a patch pipette and a Picospritzer (50 ms pulses, 7 psi; Toohey Comp. Fairfield, New Jersey, USA). Rhodamine-123 was solved in EtOH, diluted in ACSF to 50–100 *μ*M and either applied as a bolus locally or allowed to leak out from the tip of the pipette. 

Crosstalk of MitoSox and calcein fluorescence with tissue auto-fluorescence originating from lipofuscin was controlled in a subset of unstained slice cultures prior to application of the dyes. The nitric oxide (NO) donor S-Nitroso-N-acetyl-DL-penicillamine (SNAP, 100–200 *μ*M) was freshly prepared for each application and was given to the perfusion [20]. SNAP was from ALEXIS Corporation (Lausen, Switzerland); all other chemicals were from Sigma-Aldrich (Taufkirchen, Germany).

Data evaluation and 3D reconstruction were carried out with the acquisition software Andor IQ (BFIOptilas GmbH, Gröbenzell, Germany) and with ImageJ (Wayne Rasband, NIH, USA). Changes in fluorescence over time were evaluated for each fluorescence channel in manually delineated regions of interests (ROI) and are presented as Δ*f*/*f*_0_ in percent where *f*_0_ represents the average fluorescence from the first 1-2 min of a recording. 

Colocalization was analyzed after thresholding for the background noise in selected ROIs containing the vessel, by using the JACoP-plugin [21]. Pearson's and Manders' coefficients were used as a measure of colocalization where value 1 corresponds to complete colocalization and −1 or 0 to anti-colocalization for Pearson's and Manders' coefficients (*M*_1_, *M*_2_), respectively. *M*_1_ and *M*_2_ values represent the fraction of the pixels from the green fluorescence channel overlapping the red fluorescence channel and vice versa. Paired Student's two-tailed *t*-test was used for comparison between two groups. Statistical significance was defined as *P* < .05. The data are presented as mean ± S.E.M.

## 3. Results

### 3.1. Vessels in Organotypic Hippocampal Slice Cultures

By using DIC videomicroscopy, we found an elaborated network of branching voids in hippocampal slice cultures, representing the remainders of vessels down to the capillary level (~5 *μ*m diameter). The distribution closely resembled that of the hippocampus *in situ *[22] but numerous solitary vessels ending blind at both ends were also present. Larger vessels entered the hippocampus from the fissure and went along the border between the dentate gyrus and stratum lacunosum moleculare of the CA1 and CA3 giving rise to collaterals penetrating the stratum radiatum and pyramidale. In the stratum pyramidale and oriens blind ending solitary voids overwhelmed, as the branching vessels outreach the plane of cutting. Although vessels were present in the whole depth of the slice cultures (~250 *μ*m), only the vessels in the upper 50 *μ*m were used in the present study for imaging reasons. The number of vessels decreased with time in culture as described previously for slice cultures of mice [17]. Nevertheless, fragmentary vessels were still present after three weeks in culture.

### 3.2. Ca^2+^-Imaging in Perivascular and Parenchymal Astrocytes

**Figure 1:**
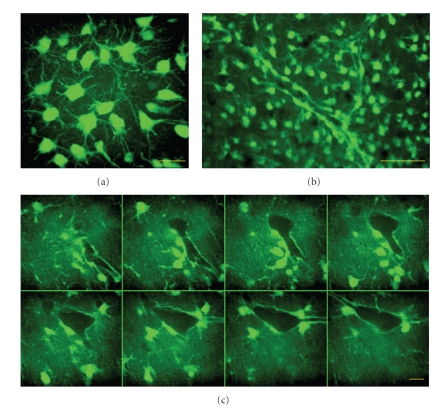
Calcein labeling of astrocytes in hippocampal slice cultures. (a) Representative 3D reconstruction of calcein labeled astrocytes in the stratum radiatum of a hippocampal slice culture after short-term (~10 min) bulk staining with calcein-AM. (b) Lower magnification (20x objective) image of calcein labeled astrocytes in the stratum lacunosum moleculare. Note the subset of astrocytes covering a large vessel. (c) Representative series of confocal images from a vessel (1.2 *μ*m steps). Calcein-labeled astrocytes and fine astrocytic processes were present in the neuropil but no calcein fluorescence could be observed within the lumen of the vessel. Astrocytic endfeet completely ensheathed the vessel. Scale bars represent 10 *μ*m in (a, c) and 100 *μ*m in (b).

**Figure 2:**
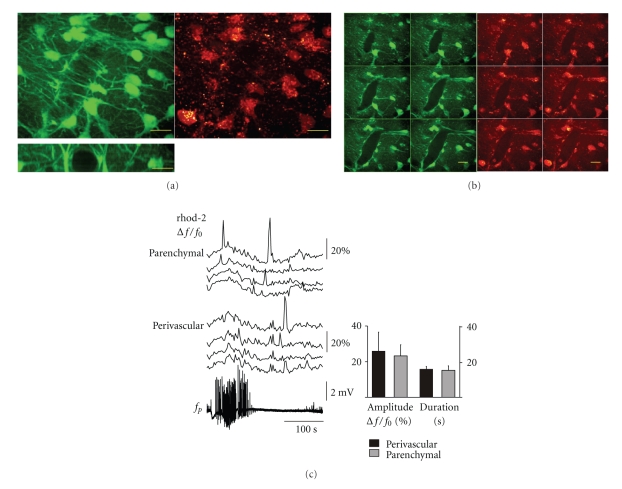
Ca^2+^-imaging in perivascular and parenchymal astrocytes. (a) 3D reconstruction of the astrocytic network covering a vessel after colabeling the slice culture with calcein-AM (green fluorescence channel) and rhod-2 AM (red fluorescence channel). Note the considerable cytosolic rhod-2 fluorescence besides the presence of the rhod-2 labeled mitochondria in the neuropil. The excerpt on the left shows the cross-section of the same vessel. (b) Z-series of confocal images (1.2 *μ*m steps) from the same vessel were used to distinguish between perivascular and parenchymal astrocytes, that is, with or without contact to the wall of the vessel. Scale bars in (a) and (b) represent 10 *μ*m. (c) Comparison of [Ca^2+^]*_i_* transients between perivascular and parenchymal astrocytes during low Mg^2+^-ACSF induced epileptiform activity. Seizure-like events (lower trace: field potential) were associated with slight elevation of astrocytic [Ca^2+^]*_i_* and were followed by large amplitude [Ca^2+^]*_i_* transients. There were no statistical differences in amplitude or duration of [Ca^2+^]*_i_* transients between perivascular and parenchymal astrocytes.

**Figure 3:**
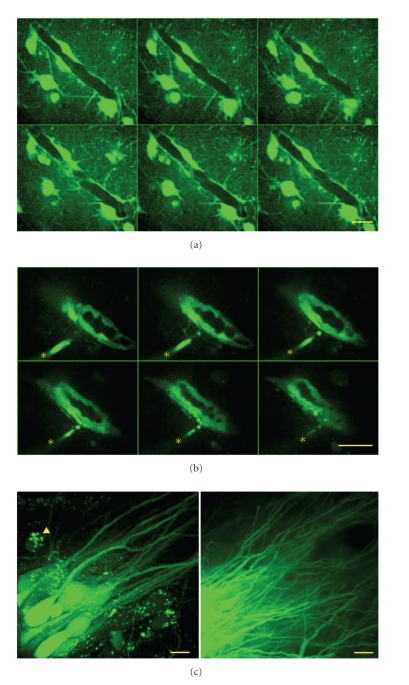
Diffusion barrier around the vessel lumen in slice cultures. (a) Z-series of confocal images (1.2 *μ*m) from a vessel after long-term (60 min) bulk staing with calcein-AM in the incubator. Note that calcein fluorescence below the astrocytic endfeet is almost absent, indicating a diffusion barrier and/or powerful extrusion mechanisms in endothelial cells. (b) Z-series of confocal images (1.2 *μ*m) from a vessel after bolus application of calcein-AM into the lumen of a vessel. Endothelial cells showed bright calcein fluorescence, whereas no fluorescence was observed in astrocytes outside of the vessel. The asterisks on the consecutive images represent the application pipette. (c) Bolus application of calcein-AM into the stratum pyramidale resulted in a neuronal/astrocytic/microglial labeling up to 80 *μ*m distance from the application place (left). Arrowhead marks a microglial cell containing calcein in vesicles. Calcein within neuronal processes can travel for several 100 *μ*m (right). Scale bars represent 10 *μ*m.

Short-term (~10 min) bulk staining of slice cultures with calcein-AM led to an almost exclusive labeling of astrocytes and microglia (Figures 1(a) and 1(b)), whereas calcein accumulation in neurons occurred only after >40 min staining. Astrocytes and microglia could be easily distinguished in the upper 50 *μ*m of the slice cultures as the latter showed filopodial movements and accumulated calcein in vesicles rather than in the cytosol unlike astrocytes (see also Figure 3(c)). Vessels were completely ensheathed by cell bodies and endfeet of astrocytes (Figure 1(c)). Endfeet often originated from astrocytes located in the parenchyma in distances of up to ~30 *μ*m. The selectivity of calcein-AM for astrocytes allowed us to compare Ca^2+^ transients in parenchymal and perivascular astrocytes by colabeling of slice cultures with the AM ester form of the calcium sensitive red fluorescent probe, rhod-2 (Figures 2(a) and 2(b)). Although rhod-2 AM accumulates in mitochondria due to its net positive charge, there is still a significant amount of dye de-esterified and captured in the cytosol [23]. Astrocytes with or without contact to the vessels (perivascular and parencyhmal) were identified prior to Ca^2+^-imaging by 3D reconstruction of the calcein-labeled astrocytic network. As an example of activity-dependent changes in astrocytic [Ca^2+^]*_i_*, low-Mg^2+^ induced epileptiform activity associated Ca^2+^ transients in astrocytes are shown in Figure 2(c). Neither the duration (15.1 ± 2.5 s versus 15.8 ± 1.4 s) nor the relative amplitude (25.9 ± 10.6% versus 23.2 ± 6.2%) of the Ca^2+^ transients were different between perivascular and parenchymal astrocytes (*n* = 48 and 52 astrocytes from 5 cultures). Occasionally, Ca^2+^ transients in parenchymal astrocytes were synchronized with the transients in perivascular astrocytes. Taken into account their strategic role in neurovascular coupling, this suggests that perivascular astrocytes translate Ca^2+^ signals from a larger astrocytic network to the vascular unit.

### 3.3. Diffusion Barrier around the Vessel Lumen in Slice Cultures

Remarkably, neither calcein-AM nor rhod-2 AM were able to stain cells below the basal membrane in slice cultures bulk stained for ~10 min. Even after one-hour staining the fluorescence of both, rhod-2 and calcein remained significantly lower within a vessel as compared with the surrounding astrocytes (Figure 3(a)). By contrast, endothelial cells and pericytes showed bright calcein labeling if calcein-AM was pressure applied into the lumen after penetration with a patch pipette (Figure 3(b)). This implicates the presence of a barrier preventing or delaying diffusion of the dye into the vessel in case of the bulk staining. 

**Figure 4:**
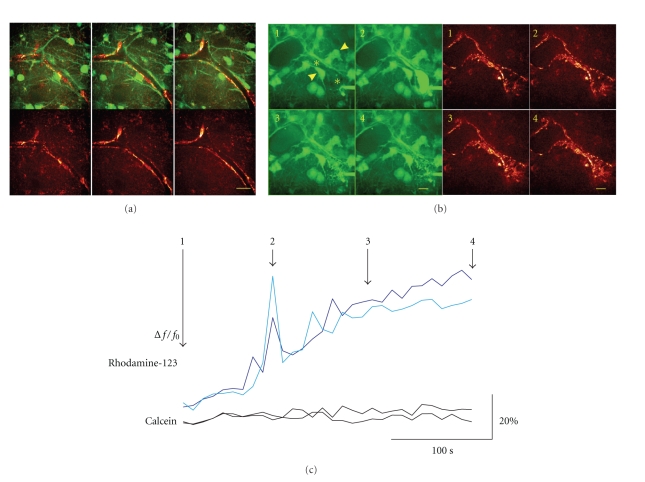
Mitochondrial free radical formation in pericytes/smooth muscle cells. (a) Representative Z-series of confocal images (1.2 *μ*m) of a vessel double labeled with calcein-AM (green fluorescence channel—upper pictures) and MitSox (red fluorescence channel). MitoSox revealed free radical formation in spindle-shaped contractile cells associated with the wall of a vessel. MitoSox was anti-colocalized with calcein in pericytes/smooth muscle cells indicating low free radical formation in astrocytic endfeet. (b) 3D reconstruction of a vessel double labeled with calcein/MitoSox. The pictures are examples taken at four time points (as marked in (c)) during bolus application of rhodamine-123 into the intraluminal space. Both, calcein and rhodamine-123 fluorescence are represented in the green fluorescence channel (left) whereas the red fluorescence channel (right) corresponds to MitoSox labeling. After penetration of the vessel with the pipette, the lumen becomes slightly fluorescent due to leakage of rhodamine-123. Intraluminal rhodamine-123 fluorescence rapidly increased during bolus application, followed by redistribution of the dye into mitochondria within the vessel. No rise in the rhodamine-123 fluorescence was observed in the surrounding astrocytes. MitoSox almost completely colocalized with rhodamine-123 revealing mitochondrial origin of free radicals in pericytes/smooth muscle cells. Note the contraction of the vessel as a consequence of the increased intraluminal pressure. Scale bars represent 10 *μ*m. (c) Changes in calcein (black traces) and rhodamine-123 (blue traces) fluorescence during bolus application of rhodamine-123 as measured in perivascular astrocytes (calcein, marked with arrowheads in (b)) and within the vessel lumen (rhodamine-123, marked with asterisks in (b)). Note that in spite of the physical contact of the astrocytic endfeet with the vessel wall, no rhodamine-123 appeared in astrocytes, further substantiating the presence of a diffusion barrier related to BBB.

Calcein-AM application into a vessel led to an immediate rise of the fluorescence within the lumen, followed by a slow redistribution into the cellular elements of the vessel within a restricted area (Figure 3(b)). By contrast, application of calcein-AM at a random location into the stratum pyramidale resulted in a widespread (>50 *μ*m) rise in fluorescence in astrocytes neurons and microglia (Figure 3(c)). In a subsequent set of experiments, we puffed a bolus of the membrane permeable mitochondrial marker, rhodamine-123 into the vessel in slice cultures previously bulk-stained with calcein-AM in the incubator (10 min). After intraluminal bolus application, rhodamine-123 fluorescence rose rapidly in mitochondria of endothelial cells and putative pericytes/SMCs (see below) but not in astrocytes adjacent to the wall of the vessel (Figures 4(b) and 4(c)). Although perforation of the vessel with the patch pipette disrupted the BBB, the leakage of rhodamine-123 from the lumen was minimal suggesting resealing of the membrane around the neck of the pipette. 

The restriction of calcein fluorescence within the boundaries of a vessel in case of bolus application and the exclusion of the dye from the vessels in case of bulk staining clearly indicated the presence of a vascular diffusion barrier related to BBB in slice cultures.

### 3.4. Vasomotility in Slice Cultures

An important observation in the previous experiments was that pressure application into the lumen invariably led to vasoconstriction, indicating the presence of contractile cells, that is, SMCs or pericytes. Fortunately, these cells could be selectively labeled with another fluorescence probe in slice cultures. Bulk staining with MitoSox, a mitochondrially targeted fluorescent probe for superoxide radicals led to intense labeling of contractile cells associated with vessels, likely SMCs and pericytes (Figures 4(a) and 4(b)). Several SMCs covered the wall of larger vessels whereas solitary spindle-shaped cells were associated with small diameter (<10 *μ*m) vessels, appearing frequently at branching points with two lengthy processes embracing the capillary. MitoSox fluorescence in SMCs and pericytes showed a dotted pattern, which is expected for a mitochondrially targeted dye. A similar dotted pattern was observed in the surrounding neuropil, but with significantly lower intensity (Figure 4(a)). In order to prove that those organelles are mitochondria, we applied a mitochondrial marker, rhodamine-123 to the immediate vicinity of the pericytes. Rhodamine-123 is a positively charged, membrane permeable fluorescent probe which can be loaded into cells via a patch pipette in cell-attached or whole cell mode and accumulates in mitochondria maintaining a negative membrane potential (~160 mV) against the cytosol [23]. After establishing the contact, MitoSox positive organelles become heavily stained with rhodamine-123 (Pearsons' coefficient: 0.5 ± 0.1, *M*_1_ (rhodamine-123 to MitoSox): 0.55 ± 0.05*M*_2_ (MitoSox to rhodamine-123): 0.69 ± 0.05) and showed typical mitochondrial movements (wiggling and directed “run and stop” sequences), thus verifying that MitoSox fluorescence originated from mitochondria (Figure 4(b)). When slice cultures were colabeled with MitoSox and calcein-AM, MitoSox was anticolocalized with calcein at the vessels (Pearson's coefficient: −0.12 ± 0.05; *n* = 14). MitoSox is an ethidium derivative, which is essentially nonfluorescent in its reduced form and its fluorescence increases when oxidized, mainly by superoxide [24]. Differences in the intensity of MitoSox fluorescence between astrocytes and putative pericytes or SMCs might represent either differences in rate of oxidation by ROS or differences in the rate of dye accumulation. Occasionally, sudden rise in MitoSox fluorescence occurred in microglial cells after more than 40 min perfusion with dye-free ACSF. This indicates that oxidation of MitoSox by ROS, rather than the accumulation of its reduced form, is responsible for the MitoSox fluorescence in our preparation. Consequently, intense MitoSox fluorescence in SMCs and in pericytes is caused by a higher mitochondrial ROS formation as compared to the surrounding astrocytes/neuropil. Differences in the Manders' coefficients *M*_1_ (calcein to MitoSox): 0.042 ± 0.006 and *M*_2_ (MitoSox to calcein): 0.34 ± 0.04 correspond to slight ROS formation in astrocytes but no calcein uptake into pericytes or SMCs.

Mechanical stimulation or increasing intraluminal pressure elicited a contraction of SMCs and pericytes leading to vasoconstriction (Figure 4(b)). On the other hand, application of the powerful vasodilatator, NO (SNAP, 100–200 *μ*M, *n* = 6) did not cause vasodilatation. This suggested that capillaries in slice cultures are maximally dilated in absence of blood flow. Nevertheless, SMCs and pericytes still retained their contractile activity for several weeks in culture, which allows the use of slice cultures as a tool for studying neurovascular coupling *in vitro*.

## 4. Discussion

In the present study, we characterized the structural and functional properties of the neurovascular unit and the BBB *in vitro *in hippocampal slice cultures. We developed fluorescence staining protocols allowing for selective labeling of different cell types of the neurovascular unit. Capillaries and vessels survived and retained their organotypic structure in culture and importantly, their lumen was segregated from the interstitium by a diffusion barrier related to BBB. Vasomotion mediated by pericytes or SMCs was also present even after three weeks in culture. Perivascular astrocytes, astrocytic endfeet, pericytes, and SMCs can be identified and selectively monitored by using our staining protocols and are accessible for electrophysiological recordings. Similarly to acute slices, pH, pO_2_, [K^+^]_o_, and [Ca^2+^]_o_ are easily manipulated in slice cultures whereas the major disadvantage of acute slices, the ongoing cell damage, is negligible after a few days in culture [19]. Thus, slice cultures offer a unique possibility to study the neurovascular unit and the BBB *in vitro*.

### 4.1. BBB in Slice Cultures

Intactness of BBB can be hardly studied in acute slices, as the preparation opens the vessels and eliminates their function as barrier [25]. By contrast, vessels reseal in slice cultures leading to formation of small enclosures of interstitial fluid. Intactness of basal lamina and the presence of tight junctional as well as transport proteins on endothelial cells were recently reported in slice cultures from mice [17, 18]. By applying calcein-AM either from the parenchymal or from the luminal side, we were able to show that these structures operate as a barrier. The BBB in slice cultures excluded calcein-AM and rhod 2-AM but not MitoSox from the vessels. The absence of calcein and rhod-2 fluorescence in endothelial cells, pericytes, and SMCs might be related to the fact that AM-esters of calcium dyes and especially of calcein are substrates of multidrug transport proteins, also expressed on the vessels in slice cultures [18, 26]. Thus slow diffusion of these dyes through the basal lamina might be counterbalanced by the activity of multidrug transport proteins at the luminal surface of BBB, finally leading to intraluminal accumulation of the nonfluorescent AM-esters.

Currently, we could not assert that tightness of BBB in slice cultures corresponds to that found *in vivo*. Nonetheless, the conditions and the cellular components necessary for the development of BBB are more close to the *in vivo* situation than in case of cocultures of endothelial cells and astrocytes [1]. Accordingly, the tightness of the artificial BBB in the combined slice culture—endothelial cell culture model, is exceedingly high [14, 15]. 

It is to note that the selectivity of calcein-AM for astrocytes in case of a short term bulk staining is characteristic for slice cultures, whereas in acute slices both neurons and glia were stained when applying the same protocol. One possible explanation might be a difference in esterase activity between neurons and astrocytes in culture. Alternatively, an up-regulation of multidrug transport proteins on neurons in slice culture might delay accumulation of calcein-AM.

### 4.2. Neurovascular Coupling and Vascular ROS Formation in Slice Cultures

Pressure application of different dyes into the lumen of a vessel revealed the presence and functional intactness of contractile cellular elements, namely, pericytes and SMCs in slice cultures. At present, we could only elicit vasoconstriction but no vasodilatation in our model. The most likely explanation is that vessels in cultures are maximally dilated in absence of blood flow and shear stress. Intraluminal dye application increases shear stress thereby leading to vasoconstriction indicating intact autoregulation of vascular tone. Alternatively, the NO-cGMP signalling pathway in pericytes/SMCs might be also altered in culture. Nevertheless, our experiments were carried out in the presence of 95% O_2_, which also favor vasoconstriction rather than vasodilatation [27]. Whether vasodilatation can be induced in preconstricted vessels awaits further investigation. 

*In vivo* studies on pericytic regulation of microcirculation have to take into account that capillaries passively follow upstream changes in blood flow [9]. The absence of blood flow is an advantage of slice cultures, since only the active contractile responses are represented by changes in capillary diameter. 

To our knowledge, this study is the first description of selective labeling of brain capillary pericytes and vascular SMCs with MitoSox. Free radical signaling is important in regulation of vasomotility [8, 28] and increased ROS formation was suggested to be involved in obstruction of microcirculation after hypoxia-reperfusion [29]. Oxygen glucose deprivation is frequently investigated in slice cultures but less attention was paid to the vascular compartment [30]. Besides their acute effects on SMCs and pericytes, oxygen glucose deprivation might cause lasting alterations of vascular function, which can be followed for weeks in culture. Understanding the mechanisms underlying free radical formation in the neurovascular unit might lead to improvement of neuroprotective strategies in stroke.

Most studies on pericytic ROS formation focus on pathological up-regulation of cytosolic NADPH oxidase activity [31]. In our preparation, mitochondria seem to significantly contribute to ROS formation in pericytes and SMCs. An interesting coincidence can be found with the study of Dai and colleagues who showed that cochlear pericytes can be selectively visualized *in vivo* by using the NO sensitive fluorescent probe DAF-2 [32]. They hypothesized that pericytes express neuronal NO synthase, and the resulting NO in addition to NO from endothelial cells leads to the intensive labeling of pericytes. Our findings offer an alternative explanation. DAF-2 fluorescence might be also influenced by increased superoxide and peroxynitrite formation, as DAF-2 reacts with oxidative derivatives of NO, rather than NO itself [20]. Consequently, the more intense labeling of pericytes with DAF-2 as compared to endothelial cells might indicate elevated ROS formation in addition to NO.

Pericytic ROS formation might also negatively interfere with the tightness and function of BBB [33]. ROS mediated deregulation of neurovascular coupling and BBB breakdown are of high clinical relevance occurring in different neurological disorders like epilepsy and Alzheimer's disease [34]. Initial BBB breakdown and subsequent angiogenesis might contribute to the progression of certain epilepsies [34, 35]. Amyloid deposits around capillaries and within degenerating pericytes were described in early onset familial Alzheimer's disease. Pericytes represent a clearance pathway for *β*-amyloid, but in turn, *β*-amyloid might impair pericytic control of vascular diameter in a free radical dependent manner [36]. An additional advantage of slice cultures is that they allow for pretreatment either with protective substances [37] or with pathogens like *β*-amyloid [30]. 

As diseases affecting the neurovascular unit seem to share some common mechanisms, future studies will take advantage of the possibility for selective monitoring of Ca^2+^-signaling in astrocytic endfeet as well as contraction and ROS formation in pericytes/SMCs.
